# AA16, a new lytic polysaccharide monooxygenase family identified in fungal secretomes

**DOI:** 10.1186/s13068-019-1394-y

**Published:** 2019-03-16

**Authors:** Camille Filiatrault-Chastel, David Navarro, Mireille Haon, Sacha Grisel, Isabelle Herpoël-Gimbert, Didier Chevret, Mathieu Fanuel, Bernard Henrissat, Senta Heiss-Blanquet, Antoine Margeot, Jean-Guy Berrin

**Affiliations:** 1 Biodiversité et Biotechnologie Fongiques, UMR1163, INRA, Aix Marseille Université, Marseille, France; 2 IFP Energies Nouvelles, 1 et 4 avenue de Bois-Préau, 92852 Rueil-Malmaison, France; 3 Plateforme d’Analyse Protéomique de Paris Sud-Ouest, Institut Micalis, UMR1319, INRA, Agro-ParisTech, Jouy-En-Josas, France; 4 UR1268, INRA, Biopolymères Interactions Assemblages, Nantes, France; 5 Architecture et Fonction des Macromolécules Biologiques, UMR7257, CNRS, Aix Marseille Université, Marseille, France; 6 USC1408, INRA, Architecture et Fonction des Macromolécules Biologiques, Marseille, France

**Keywords:** Filamentous fungi, Plant biomass, Cellulolytic enzymes production, LPMO, Biofuels

## Abstract

**Background:**

Lignocellulosic biomass is considered as a promising alternative to fossil resources for the production of fuels, materials and chemicals. Efficient enzymatic systems are needed to degrade the plant cell wall and overcome its recalcitrance. A widely used producer of cellulolytic cocktails is the ascomycete *Trichoderma reesei*, but this organism secretes a limited set of enzymes. To improve the saccharification yields, one strategy is to upgrade the *T. reesei* enzyme cocktail with enzymes produced by other biomass-degrading filamentous fungi isolated from biodiversity.

**Results:**

In this study, the enzymatic cocktails secreted by five strains from the genus *Aspergillus* (*Aspergillus japonicus* strains BRFM 405, 1487, 1489, 1490 and *Aspergillus niger* strain BRFM 430) were tested for their ability to boost a *T. reesei* reference cocktail for the saccharification of pretreated biomass. Proteomic analysis of fungal secretomes that significantly improved biomass degradation showed that the presence of proteins belonging to a putative LPMO family previously identified by genome analysis and awaiting experimental demonstration of activity. Members of this novel LPMO family, named AA16, are encountered in fungi and oomycetes with life styles oriented toward interactions with plant biomass. One AA16 protein from *Aspergillus aculeatus* (AaAA16) was produced to high level in *Pichia pastoris.* LPMO-type enzyme activity was demonstrated on cellulose with oxidative cleavage at the C1 position of the glucose unit. AaAA16 LPMO was found to significantly improve the activity of *T. reesei* CBHI on cellulosic substrates.

**Conclusions:**

Although *Aspergillus* spp. has been investigated for decades for their CAZymes diversity, we identified members of a new fungal LPMO family using secretomics and functional assays. Properties of the founding member of the AA16 family characterized herein could be of interest for use in biorefineries.

**Electronic supplementary material:**

The online version of this article (10.1186/s13068-019-1394-y) contains supplementary material, which is available to authorized users.

## Background

Lignocellulosic biomass is a renewable and abundant feedstock, considered as a promising alternative to non-sustainable fossil resources for the production of biofuels, biomaterials and bio-based chemicals [1, 2]. Its transformation requires an extensive deconstruction of the plant cell wall polymers, namely the cellulose and hemicellulose polysaccharides, that are intermeshed together with lignin to form a highly resistant structure. Efficient hydrolytic enzyme cocktails are, therefore, required but are still at the present time a major bottleneck for cost-effective industrial processes.

The degradation of plant polysaccharides into simple sugars can be achieved using enzymes secreted by biomass-degrading organisms such as bacteria and filamentous fungi. These carbohydrate-active enzymes (or CAZymes) are classified in the CAZy database (http://www.cazy.org/) into several families based on their amino acid sequence similarities [3]. Cellulases and hemicellulases, which belong to the glycoside hydrolases (GH), have been studied for more than 60 years for their ability to depolymerize cellulose and hemicelluloses. More recently, novel enzymes called lytic polysaccharide monooxygenases (LPMOs) were discovered [4] and raised a high interest due to their ability to boost the hydrolysis of lignocellulosic biomass [5]. LPMOs are able to cleave glycosidic bonds using an oxidative mechanism, in the presence of oxygen atoms (coming from dioxygen or hydrogen peroxide) and an extracellular electron donor [6, 7]. Electrons can be provided by small organic compounds such as lignin fragments and plant- or fungus-derived phenols, by enzymatic systems such as cellobiose dehydrogenase (CDH) or by photocatalytic systems [8]. All LPMOs share some common features, such as a copper-containing active site, in which the metal is coordinated by three nitrogen atoms from two histidine side chains and the N-terminal amine group that form the so-called “histidine brace” [9, 10].

LPMOs are currently grouped into six CAZy auxiliary activities (AA) families (AA9–AA11, AA13–AA15), based on a bioinformatics analysis of their amino acid sequences similarities. These families are found in several taxonomic groups, and have been shown to be active on numerous substrates. The fungal AA9 family includes enzymes active on cellulose and cello-oligosaccharides, but also polysaccharides containing β-1,4-linked glucose units such as xyloglucans, glucomannans and β-glucans [11–13]. LPMOs from the AA10 family, which are found mainly in bacteria and some viruses, are active on both chitin and cellulose [14]. AA11 and AA13 families are found exclusively in fungi; only a few of their members, which are, respectively, active on chitin and starch components, have been characterized so far [15–17]. The fungal AA14 family was discovered recently, and two of its members showed activity on recalcitrant xylan coating cellulose fibers [18]. Finally, the discovery in 2018 of the AA15 family revealed the existence of LPMOs of animal origin (invertebrates), active on both cellulose and chitin [19]. These enzymes have only recently been described, and the discovery of more families and new enzyme specificities can be expected.

Cellulases, hemicellulases and LPMOs are key components of industrial cocktails dedicated to biomass degradation [20]. The vast majority of these cocktails are based on enzymes secreted by *Trichoderma reesei*, a fungal species that is known for its efficient cellulolytic enzymes secretion, and benefits from decades of strain improvement [21, 22]. However, the sequencing of its genome revealed a surprisingly small number of genes involved in polysaccharides degradation [23, 24], and several missing accessory activities [25]. With only three AA9 LPMOs encoded in its genome, *T. reesei* has one of the smallest sets of LPMOs among fungal saprotrophs, which are usually rich in AA9 with up to 40–50 genes [26].

To compensate this lack of diversity, which may be a bottleneck for *T. reesei* cocktails improvement, one strategy is to upgrade *T. reesei* cocktails with enzymes from other biomass-degrading filamentous fungi isolated from biodiversity. Such enzymes can be found by mining the sets of secreted enzymes (i.e, secretomes) produced by fungal strains on various carbon sources, thanks to the development of protein identification tools such as LC–MS/MS and the increasing number of available annotated fungal genomes [27, 28]. Since a large panel of CAZymes and LPMOs are differentially secreted by fungal saprotrophs [29, 30], comparative secretomics is a promising approach to discover novel enzymes playing a role in plant biomass deconstruction. Herein, we report the identification of a new LPMO family among *Aspergillus* spp. secretomes improving the conversion of plant biomass. One member of this new LPMO family, termed AA16, was characterized in terms of substrate specificity, regioselectivity of oxidation and contribution to cellulose degradation.

## Results

### Exploration of fungal secretomes to improve biomass saccharification

During previous screenings of fungal strains for their biomass-degradation ability, several strains of the *Aspergillus* genus appeared promising [31]. In the present study, four strains of *Aspergillus japonicus* and one strain of *Aspergillus niger* were grown in the presence of three inducers [maize bran (MB), sugar beet pulp (SBP) and cellulose (Avicel)] to favor the secretion of diverse lignocellulose-active enzymes. After 7 days of growth, the 15 different secretomes (Additional file 1: Table S1) were tested for their ability to improve a reference *T. reesei* cocktail obtained from strain CL847 [32, 33] for the saccharification of three dilute-acid steam-exploded biomasses: wheat straw, Miscanthus and poplar.

**Fig. 1:**
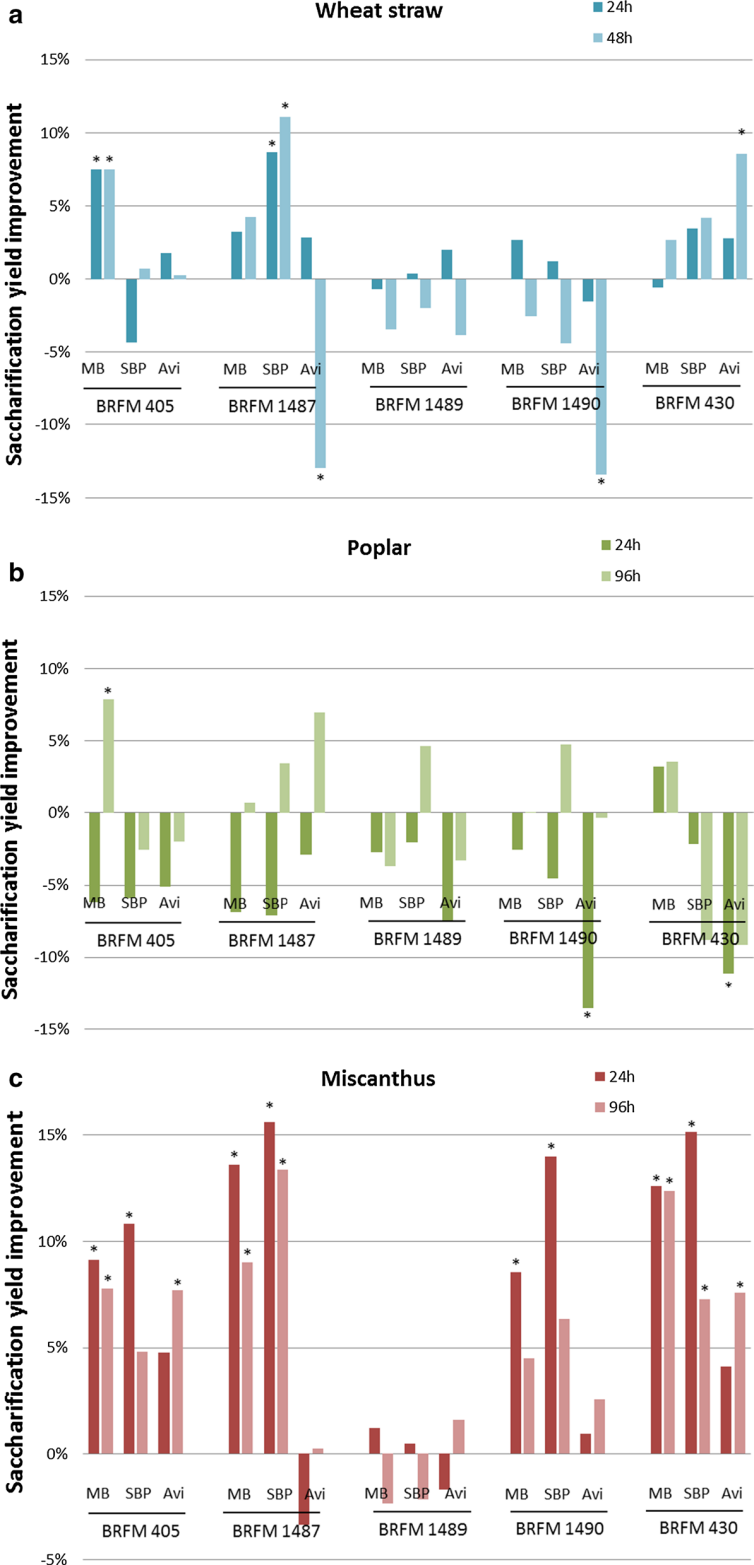
Biomass saccharification performances of 15 *Aspergillus* spp. secretomes. Effect on the saccharification of pretreated biomass (5% w/v) of the addition to a *T. reesei* cellulolytic cocktail of *Aspergillus* secretomes produced by strains BRFM 405, 430, 1487, 1489 and 1490 on maize bran (MB), sugar beet pulp (SBP) and Avicel (Avi). **a** Wheat straw; **b** poplar; **c** miscanthus. The bars show the improvement of cellulose conversion yields in the presence of secretomes compared to cellulose conversion yields obtained with *T. reesei* cocktail alone, after 24 h and 96 h of reaction. Means were calculated on more than 10 replicates; the * indicates a Student test *p*-value lower than 0.05

After 48 h of biomass saccharification, three secretomes (produced by *A. japonicus* BRFM 405 on MB, *A. japonicus* BRFM 1487 on SBP and *A. niger* BRFM 430 on Avicel) were able to significantly improve the cellulose conversion yield of wheat straw (Fig. 1a), which is noteworthy given that the yields using the reference cocktail alone were already high, reaching 60% glucose release from cellulose after only 24 h, and over 90% at the plateau (Additional file 1: Figure S1). The *T. reesei* cocktail was less efficient on poplar (60% of maximum saccharification yield, see Additional file 1: Figure S1), and this woody biomass proved to be also recalcitrant to the *Aspergillus* secretomes : only one of them (produced by *A. japonicus* BRFM 405 on MB) was able to significantly improve the yield after 96 h of reaction (Fig. 1b).

Concerning Miscanthus, a total of 10 secretomes among the 15 tested were able to significantly improve the cellulose conversion yield (Fig. 1c), after 24 h and/or 96 h of reaction. Interestingly, the secretomes of *A. japonicus* BRFM 405 and *A. niger* BRFM 430 produced on all of the 3 inducers were able to boost Miscanthus saccharification, while none of the secretomes of the *A. japonicus* BRFM 1489 strain had an effect on yield. As for the two remaining strains (*A. japonicus* BRFM 1487 and 1490), their secretomes produced on lignocellulosic substrates (MB and SBP) improved the glucose yield by 9 to 16%, while their secretomes produced on Avicel did not lead to any significant improvement. This demonstrates that not only the strain, but also the inducer has an effect on the biomass degradation ability of the produced enzyme cocktails. Concerning the time effect, it can be noticed that all secretomes produced on MB and SBP have a lower effect after 96 h of reaction than after 24 h, which can be explained by the fact that the higher the yield, the more it becomes difficult to improve it when approaching the saccharification plateau. The secretomes of strains BRFM 405 and 430 produced on Avicel show the opposite effect, with a greater yield improvement at 96 h, which could mean that the enzymes responsible for the boost act more slowly than those of other secretomes. Overall, these results show that several *Aspergillus* secretomes are able to improve biomass saccharification, and that the enzymes responsible for the boost are probably different from one secretome to another.

### Comparative proteomic analysis of fungal secretomes

To understand the differences in terms of enzyme composition between these fungal secretomes, proteomic analyses were performed by liquid chromatography coupled to tandem mass spectrometry (LC–MS/MS), and the detected peptides were assigned using the public genomes of *A. niger* and *A. aculeatus*. This latter species is very close to *A. japonicus*, and the two species are identical in some classifications [34], which explains our decision to use the *A. aculeatus* genome to identify and annotate *A. japonicus* proteins.

**Fig. 2:**
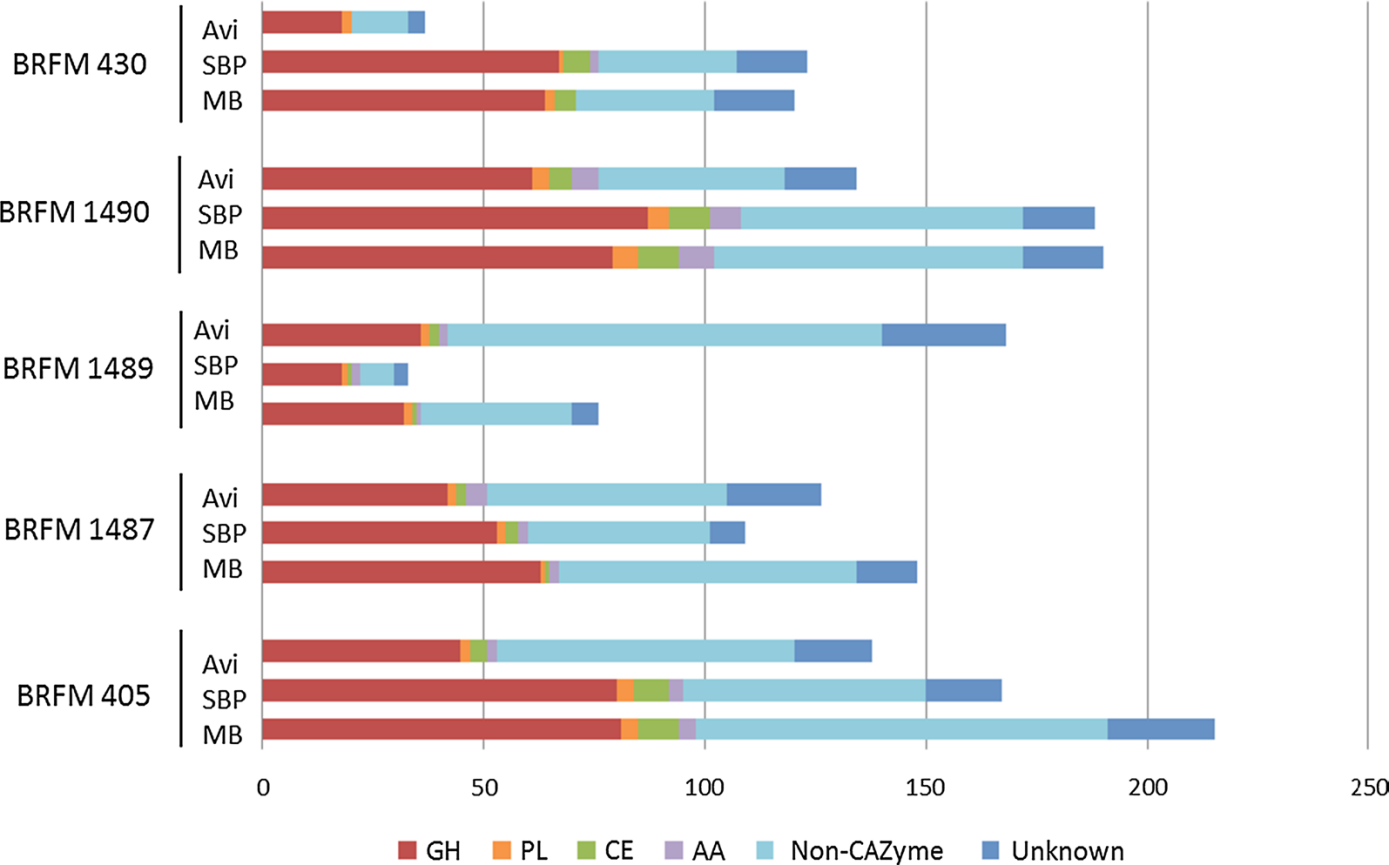
Protein content of the *Aspergillus* spp. secretomes. Composition of the 15 secretomes in number of identified CAZymes (*GH* glycoside hydrolases, *PL* polysaccharide lyases, *CE* carbohydrate esterases, *AA* auxiliary activities), non-CAZyme proteins and proteins of unknown function

The number of different proteins present in each secretome is highly variable, ranging from 33 to over 200. Most of the produced secretomes have a larger diversity of enzymes than the CL847 reference cocktail, which contains approximatively 30 proteins. However, it must be noted that this *T. reesei* cocktail was produced under different conditions than the *Aspergillus* secretomes. As expected, the *Aspergillus* secretomes are rich in CAZymes, accounting for 25–67% of the total number of proteins, but they also contain enzymes from other classes (oxidases, esterases, proteases, nucleases, etc.) and proteins of unknown function (Fig. 2).

The composition of each secretome that showed an improvement of saccharification was further examined, with special attention for proteins of putative or unknown function. Among other proteins, our attention was dragged to a protein of unknown function (ortholog of *A. aculeatus* XP_020060743.1), which was found only in MB and SBP secretomes of *A. japonicus* BRFM 1490 that increased Miscanthus saccharification (Fig. 1c). Interestingly, orthologs of this protein are not found in the *T. reesei* genome. As revealed by sequence analyses, this protein displays some common structural features with known LPMOs. For instance, its N-terminal residue after signal peptide cleavage is a histidine residue. A deeper analysis of the mass spectrometry data obtained from *A. japonicus* BRFM 1490 secretome revealed that the N-terminal peptide displays a methylated histidine (mass + 14.0157 Da), a feature commonly observed in fungal LPMOs.

### Bioinformatic analysis of a new LPMO family

The protein of unknown function (ortholog of *A. aculeatus* XP_020060743.1) identified in the fungal secretomes is part of a putative LPMO family that has been reported in a genome mining approach using a HMM model developed by Voshol et al. [35]. Without any experimental demonstration of activity, this family could not be added to the CAZy database, which contains only biochemistry-based families. In the absence of biochemical characterization, this family was temporarily called X273.

**Fig. 3:**
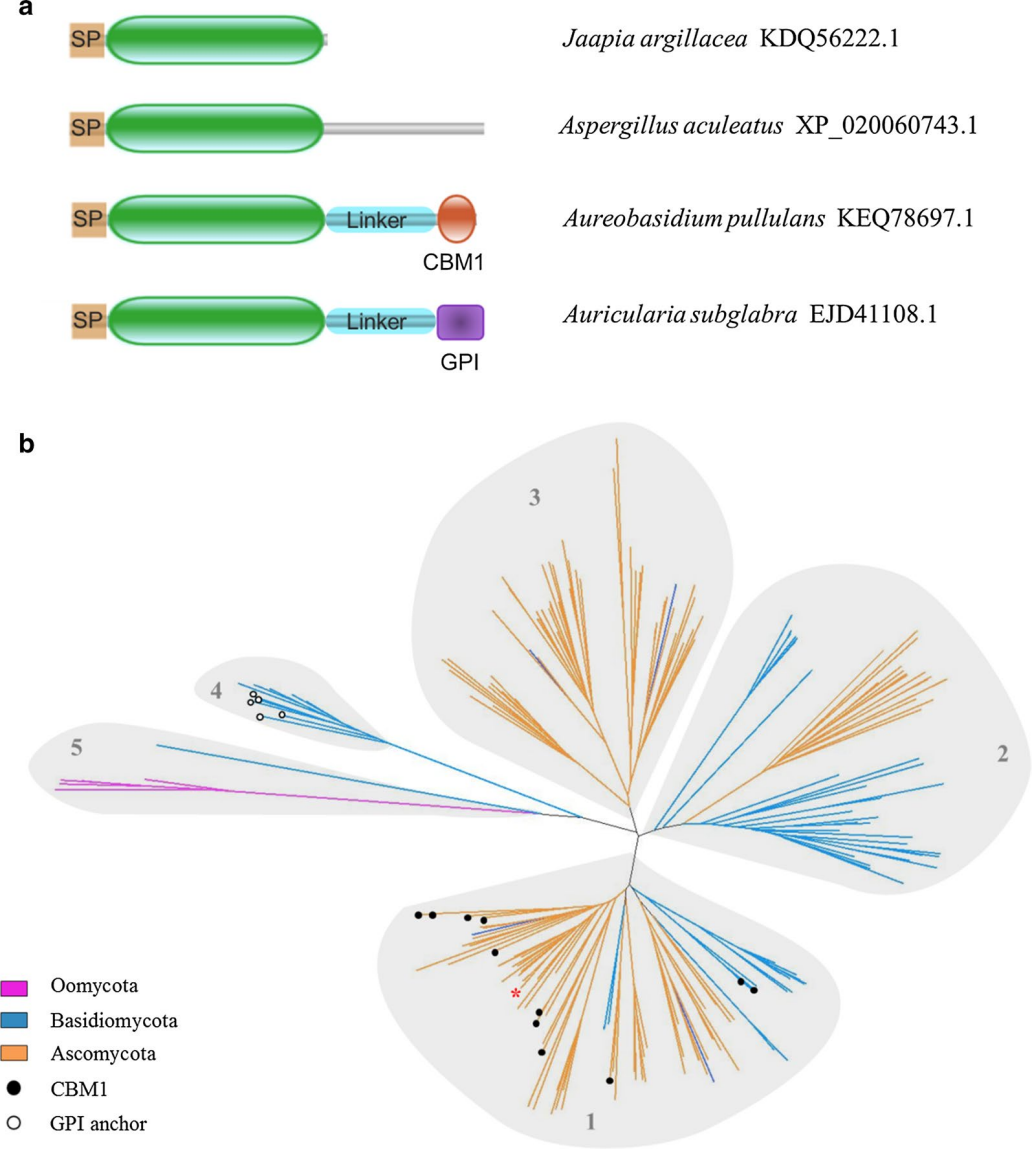
Phylogeny and modularity of the new LPMO family. **a** Graphical representation of the different types of modularity existing among the family, citing examples of proteins form various species with their Genbank ID. The catalytic module, shown in green, is preceded by a signal peptide (SP), and can be followed by a C-terminal extension, a CBM1 module or a GPI anchor. **b** Radial phylogram of 208 selected sequences, based on the alignment of their catalytic module alone. Edges are colored according to the taxonomic division of the corresponding organisms. Full circles indicate CBM1-containing sequences and empty circles indicate GPI anchor-containing sequences. The sequence from *Aspergillus aculeatus* is indicated by a red star

An analysis of non-redundant protein database (in August 2018) revealed a total of 1065 sequences containing a X273 module, belonging to 580 microorganisms presenting a variety of life styles oriented toward interactions with plant biomass: a majority of the sequences belong to saprophytes and phytopathogens, but plant endophytes and symbionts are also represented. These organisms are mainly fungal (with about 80% of Ascomycetes, 18% of Basidiomycetes and a few Chytridiomycetes), but X273 modules are also found in some Oomycetes, which are fungus-like protists, and more specifically in known phytopathogens of the genera *Phytophthora* and *Pythium*. The Oomycetes present a slight family expansion, with an average number of 5.7 X273 genes per species compared to 1.8 for the Fungi. The X273 module is preceded in all these sequences by a signal peptide, and is often followed by a C-terminal extension of varying length. In a few cases, this extension is composed of a linker and a CBM1 module or a glycosylphosphatidylinositol (GPI) anchor (Fig. 3a).

The sequences of 208 X273 modules (edited to remove the CBM1, C-terminal extension or GPI anchor) from 107 organisms representative of major taxons were aligned and used to build a phylogenetic tree (Fig. 3b), in which five major clades can be distinguished. Clades 1 and 2 group together enzymes from Ascomycetes and Basidiomycetes that have likely been kept from a common ancestor of these two phyla. Conversely, clade 3 gathers only Ascomycetes sequences (except two sequences from *Cryptococcus* sp. that could have been acquired by horizontal gene transfer [36]) and clade 4 consists of Basidiomycetes sequences only, indicating that these sequences evolved after the split between these two phyla. Finally, clade 5 displays sequences from Oomycetes and one sequence from the basidiomycete *Cronartium quercuum*, which could mean that these sequences were kept from a common ancestor of Oomycetes and Fungi, or could also be the result of a horizontal gene transfer. Interestingly, the sequences belonging to proteins containing a CBM1 or a GPI anchor are not spread across the tree but are gathered, respectively, in clades 1 and 4, suggesting that the X273 modules of these groups may have evolved for distinct functions.

Sequence alignment of the modules reveals several well-preserved regions within the family (Additional file 1: Figure S2). All sequences include an N-terminal histidine, as well as a second strictly conserved histidine, which is a characteristic of the copper binding site (Histidine brace) of all LPMOs characterized so far. There are about twenty other highly conserved residues, including a conserved Q-T/N-Y motif that fits the N/Q/E-X-F/Y motif identified by Vu et al. [37] in families AA9, AA10, AA11 and AA13, containing the tyrosine or phenylalanine residue taking part in the coordination of copper in the active site.

### Heterologous expression and purification

The X273 protein of *A. aculeatus* (Genbank ID XP_020060743.1) was produced in *Pichia pastoris* without its C-terminal extension. The recombinant protein bearing a C-terminal polyhistidine-tag was first produced in flasks in the presence of trace metals including copper and purified from the culture supernatant by immobilized metal ion affinity chromatography (IMAC), following the same protocol used for AA9 LPMOs [11]. The produced protein had a size of ~ 38 kDa, higher than the expected 21 kDa, attributable to *N*- and/or *O*-glycosylation. N-terminal sequencing revealed that in approximately 30% of the sample, the protein was deprived of the N-terminal residue, an heterogeneity that is frequently encountered in the production of recombinant proteins and could be due to peptide bond cleavage by locally produced reactive oxygen species [38, 39]. The rest of the sample did not contain the expected N-terminal histidine, which was replaced either by an aspartate residue or by other non-identified modifications. Recent reports have shown that in LPMOs, the residues surrounding the copper are particularly prone to oxidation [7, 40]. The N-terminal histidine of *A. aculeatus* X273 could have been modified into aspartate, 2-oxo-histidine or other intermediate products due to metal-catalyzed oxidation in the presence of copper as described in [41–43], most probably leading to an inactive enzyme.

Alternatively, the same recombinant X273 protein was produced in a bioreactor in the presence of trace metals including copper. A glycerol batch phase was followed by an induction fed-batch phase using methanol (Additional file 1: Figure S3). An aliquot of the culture supernatant was purified by IMAC, to reach a final purified protein production yield of 0.5 g per liter of culture. N-terminal sequencing showed the expected sequence with an intact N-terminal histidine in over 90% of the produced protein, while the cleaved sequence represented only less than 10%.

### Substrate specificity and regioselectivity of cleavage

Preliminary enzyme activity assays were performed using the *P. pastoris* bioreactor supernatant that contains a major band at ~ 38 kDa and the purified *A. aculeatus* X273 on phosphoric acid swollen cellulose (PASC). The soluble degradation products were characterized using high-performance anion-exchange chromatography coupled with amperometric detection (HPAEC–PAD), and the assay carried out using the bioreactor supernatant revealed an important release of soluble cello-oligosaccharides (DP2-DP6, see Additional file 1: Figure S4), which was a good starting point for further investigations. However, the protein purified by IMAC displayed reduced activity on cellulose, even tested at high protein loading (Additional file 1: Figure S4), suggesting that the purification using the standard IMAC column was detrimental for the activity of this enzyme. Therefore, the protein was purified to homogeneity by ion exchange chromatography (Additional file 1: Figure S5). ICP-MS analysis was used to confirm the presence of copper in the active site, with ~ 1 copper atom per protein molecule. The purified X273 protein from *A. aculeatus* was able to produce hydrogen peroxide without substrate in the presence of l-cysteine and ascorbate (Additional file 1: Figure S6), which is a common feature for LPMOs [44].

**Fig. 4:**
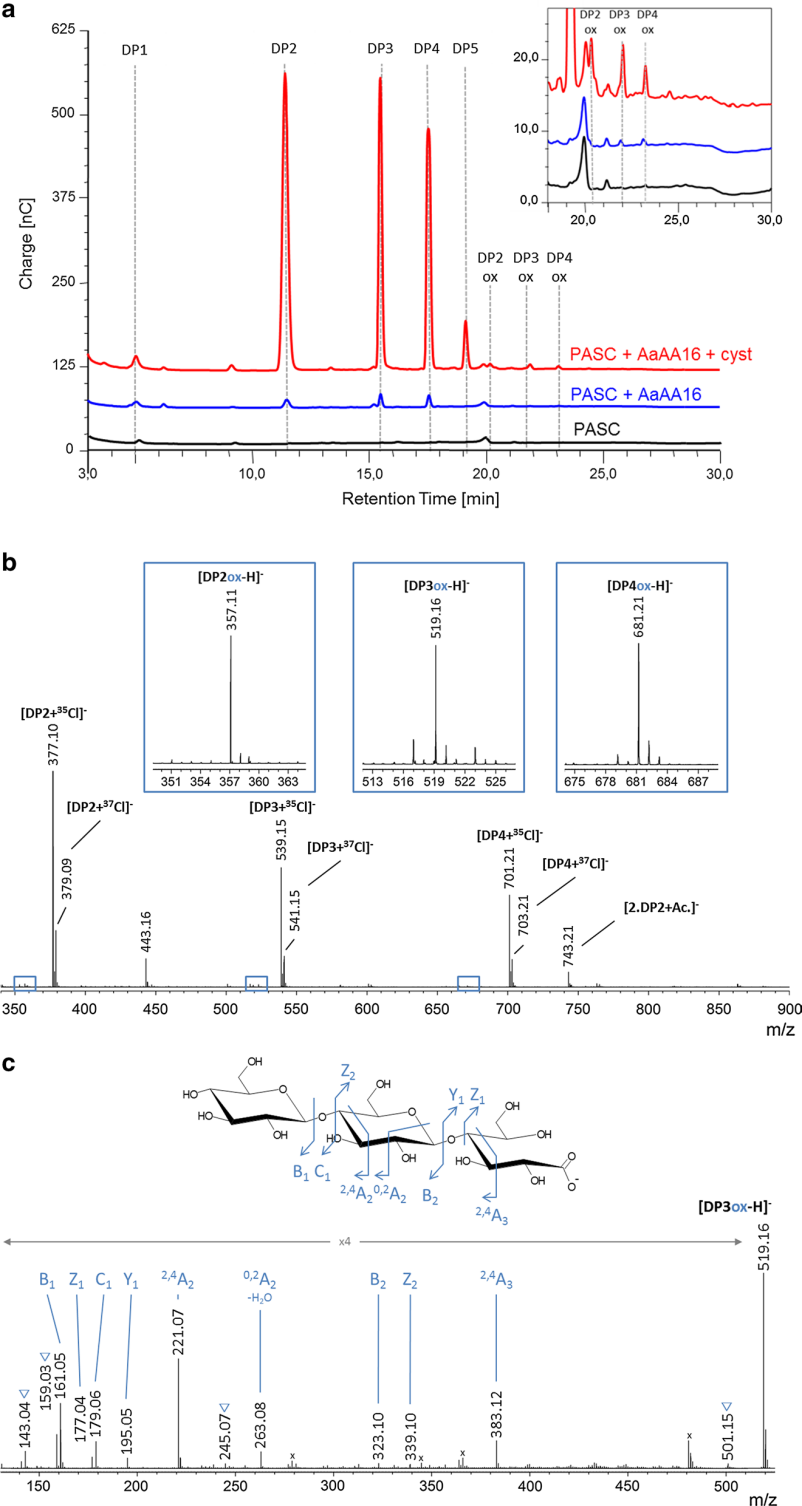
Oxidative cleavage of cellulose by AaAA16. **a** HPAEC–PAD chromatograms showing soluble products generated from 0.1% PASC cellulose using 4.4 µM AaAA16, with or without l-cysteine (1 mM). The peak annotations are based on comparison with oligosaccharides standards, native (DP1–DP5) or oxidized at the C1 position (DP2ox-DP4ox). The box at the right top shows an enlargement of the C1-oxidized product region. **b** MS spectrum of soluble degradation products generated by AaAA16 from PASC. Mass spectrometry analysis of soluble degradation products generated by AaAA16 from PASC. The main panel shows the full-scale spectrum of the sample containing native and oxidized cello-oligosaccharides, and the boxed regions are magnified to show the peaks corresponding to oxidized cello-oligosaccharides. **c** MS/MS spectrum of the DP3 oxidized product peak (m/z 519.16) observed on (**b**). The fragmentation pattern corresponds to a C1 oxidized species with an aldonic acid at the reducing end. Observed fragments are depicted on the structure. Blue triangles: Water losses. Black stars: contaminant peak from an ion co-isolated during the MS/MS precursor selection

Further enzymatic assays were performed at moderate enzyme loading on several substrates containing β-linked glucose units [phosphoric acid swollen cellulose (PASC), microcrystalline cellulose (Avicel), β-glucan, glucomannan] as well as cello-, xylo- and xyloglucan-oligosaccharides. Significant activity was detected on cellohexaose (Additional file 1: Figure S7) and cellulosic substrates (Fig. 4a) with the release of a majority of non-oxidized products (DP2–DP5), and small peaks eluting at the same retention time as C1-oxidized products (DP2ox–DP4ox). Mass spectrometry was used to confirm the presence of oxidized oligosaccharides at a mass of m/z + 16 compared to the non-modified oligosaccharides (Fig. 4b). These peaks could correspond either to an aldonic acid form (C1-oxidized) or a gemdiol form (C4-oxidized), but the ketone form, which is usually observed at m/z − 2 in the case of a C4-oxidation was not found. The MS/MS analysis of the DP3-oxidized product, taken as an example, was consistent with the presence of an oxidation at the reducing end, providing further evidence that the X273 protein from *A. aculeatus* is a C1-oxidizing LPMO active on cellulose (Fig. 4c).

Altogether, the results suggest that the characterized protein is a copper-containing enzyme able to produce hydrogen peroxide in the absence of substrate and able to oxidatively cleave cellulose with C1 regioselectivity. The enzymatic characterization of *A. aculeatus* enzyme establishes that the enzyme has LPMO activity, and therefore, family X273 was renamed AA16 in the CAZy database. Consequently, *A. aculeatus* X273 is named AaAA16.

### Synergy assays

**Fig. 5:**
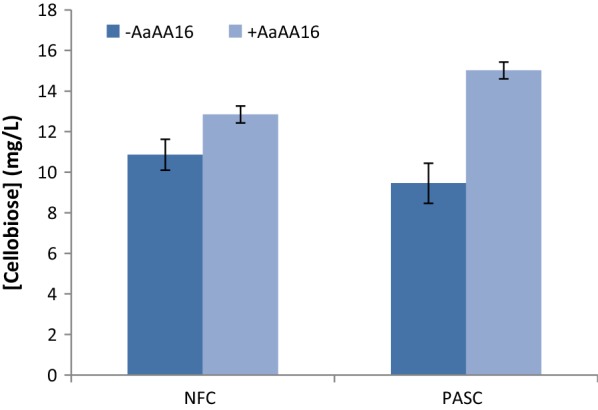
Synergy between AaAA16 and CBHI for the degradation of cellulosic substrates. Cellobiose released from 0.1% PASC or NFC by *T. reesei* CBHI (1 mg/g of substrate), with or without prior 24-h treatment by AaAA16 (10 mg/g), was quantified by HPAEC–PAD. Error bars represent the standard deviation calculated on three replicates

Many LPMOs are able to act in synergy with cellulases and in particular with cellobiohydrolase I (CBHI) [5, 45–48], which is mainly responsible for the degradation of crystalline cellulose [49]. To determine whether it is the case of the AaAA16 enzyme, a sequential assay was performed on nano-fibrillated cellulose (NFC) and PASC. The cellulosic substrates were first treated for 24 h with AaAA16 in the presence of l-cysteine; the control condition contained l-cysteine but no AA16 enzyme. After inactivating enzymes by boiling, the soluble products were removed and the insoluble fraction was washed. *T. reesei* CBHI was then added for 2 h, and the produced cellobiose was quantified by HPAEC–PAD. As shown in Fig. 5, the product released from both cellulosic substrates was more important after AaAA16 treatment than in the condition without prior AA16 treatment, probably due to the new chain ends produced by LPMO action that allowed an increased CBHI activity. *T. reesei* CBHI being a major component of industrial cellulolytic cocktails, the ability of AaAAxx to improve its activity on cellulose is an interesting feature indicating that the new AAxx family could be explored for biotechnological applications.

## Discussion

The *A. aculeatus* AA16 LPMO characterized in this study shares many features with fungal LPMOs from other families, including (i) two strictly conserved histidines forming the copper histidine brace, (ii) methylation of the first histidine residue in fungal secretomes, (iii) the presence of one copper atom per protein and production of hydrogen peroxide (iv) ability to cleave cellulose and cello-oligosaccharides while producing C1-oxidized products, (v) synergy with CBHI for the degradation of cellulose. Although its sequence and its modularity have long suggested that the family may have LPMO activity, none of its members has been biochemically characterized so far, possibly due to recombinant production and handling problems. AaAA16 is indeed a challenging enzyme and we faced difficulties in obtaining a functional protein due to self-oxidation of the recombinant enzyme produced in flasks and inactivation during the affinity chromatography purification. Oxidation of the N-terminal histidine of LPMOs is a frequently encountered issue due to the formation of reactive oxygen species at the active site in the presence of copper. Recently, it was hypothesized that the methylation of this residue in fungi could be a protective mechanism against self-inactivation of the enzyme [50]. This post-translational modification is lacking in *P. pastoris*, which could lead to consider the use of another host organism such as *A. niger* for recombinant expression of AA16 enzymes. However, in the case of AaAA16, we managed to circumvent this problem with the set-up of a bioreactor production which led to a functional enzyme.

Another noticeable feature of this new LPMO is its atypical product profile, which differs from those of other fungal LPMOs active on cellulose. Indeed, the ratio of C1-oxidized vs. non-oxidized products is much lower than those of so far characterized AA9 enzymes, suggesting that the two families may have a different reactivity. More generally, the differences existing between AA9 and AA16 enzymes are of interest, considering that these two fungal LPMO families seem to be targeting the same substrate. It can be noted that the gene expansion of the AA16 family, with an average of 5.7 sequences per genome for Oomycetes and 1.8 for Fungi, is strikingly lower than in the AA9 family, many fungal species having over 30 AA9 genes in their genome [26]. Moreover, cellobiose dehydrogenase (CDH), which is a well-established enzymatic redox partner of AA9 LPMOs, was not found in the *A. japonicus* BRFM 1490 secretomes. Interestingly, in vitro assays with a recombinant CDH from *Podospora anserina* did not seem to promote AA16 activity (Additional file 1: Figure S8). This observation could reveal a difference in the activation of this LPMO family compared to AA9 LPMOs. The secretomic analysis also revealed that two AA9 enzymes, including one with a CBM1, are co-secreted with *A. japonicus* AA16, which could indicate that they have a different biological role or a possible synergistic effect.

## Conclusions

Using a comparative secretomic approach, we were able to identify and characterize the founding member of a new LPMO family that plays a role in plant biomass deconstruction, and could be of interest for biotechnological applications. Although *Aspergilli* species have been deeply investigated for decades and a large number of their CAZymes have been characterized, the activity of this new LPMO family had not been unveiled until now.

To better understand the action of members of this new family on plant biomass and their possible biological roles, it will be necessary to characterize other AA16 enzymes, including some multi-modular enzymes bearing a CBM1 or a GPI anchor. Further structural and spectroscopic investigations are also required to provide insights into the mechanism of the AA16 family.

## Materials and methods

### Preparation of secretomes

The *Aspergillus japonicus* and *Aspergillus niger* strains used in this study are maintained in the collection of the “Centre International de Ressources Microbiennes—Champignons Filamenteux” (CIRM-CF, INRA, Marseille, France; http://www.inra.fr/crb-cirm/) under accession numbers BRFM 405, 430, 1487, 1489 and 1490. They were grown on agar medium containing 20 g L^−1^ malt extract and 1 g L^−1^ yeast extract.

Liquid culture media containing sugar beet pulp or an autoclaved fraction of maize bran (supplied by ARD, Pomacle, France) as carbon sources and protein secretion inducers were prepared as follows: 15 g L^−1^ inducer; 2.5 g L^−1^ maltose; 1.84 g L^−1^ diammonium tartrate as nitrogen source; 0.5 g L^−1^ yeast extract; 0.2 g L^−1^ KH_2_PO_4_; 0.0132 g L^−1^ CaCl_2_·2H_2_O and 0.5 g L^−1^ MgSO_4_·7H_2_O. Another inducing medium was prepared using 4 g L^−1^ Avicel (Sigma-Aldrich, Saint-Quentin Fallavier, France); 10 g L^−1^ xylose; 1.8 g L^−1^ diammonium tartrate; and the same amounts of yeast extract and minerals as before. The three culture media were inoculated with 2.10^5^ spores/mL for each of the five strains, and incubated in the dark in baffled vials, at 30 °C under rotary agitation at 105 rpm (Infors, Massy, France).

After 7 days of incubation, the cultures were stopped and the liquid medium was separated from the mycelium using Miracloth (Merck-Millipore, Darmstadt, Germany). Each secretomes (40 mL) was filtered on a 0.22-µm polyether sulfone membrane (Merck-Millipore) and diafiltered on a polyether sulfone membrane with a 10-kDa cut-off (Vivaspin, Sartorius, Göttingen, Germany) with 50 mM sodium acetate buffer pH 5.2 to a final volume of 2 mL. The secretomes were stored at − 20 °C until use. Their total protein concentration was evaluated by the Bradford (Protein Assay, BioRad, Ivry, France) and BCA (Bicinchoninic Acid Protein Assay, Sigma-Aldrich) methods using a bovine serum albumin (BSA) standard.

### LC–MS/MS protein identification

For each secretome, 15 µg of proteins was loaded on a 10% Tris–glycine precast SDS-PAGE gel (Mini-PROTEAN TGX, BioRad). After a short migration (0.5 cm) in the stacking gel, the gels were stained with Coomassie blue (BioRad) and each electrophoresis track was cut into two 2-mm-wide strips. Proteomic identification was performed at the Plate-forme d’Analyse Protéomique de Paris Sud-Ouest (PAPPSO, INRA, Jouy-en-Josas, France; http://pappso.inra.fr/), according to a protocol described in [51]. Briefly, the digestion of the proteins contained in the gel strips was carried out according to a standard trypsinolysis process, using modified trypsin (Promega, Charbonnières-les-Bains, France). Peptide analysis was performed by Ultimate 3000 RSLCnano liquid chromatography (Thermo Fisher Scientific, Waltham, Massachusetts, USA) coupled to a Q-exactive mass spectrometer (Thermo Fisher Scientific) using electrospray ionization. Peptide attribution and protein annotation were performed by comparing mass spectrometry data to predicted proteins in the genomes of *Aspergillus niger* (http://www.uniprot.org/) and *Aspergillus aculeatus* (https://genome.jgi.doe.gov/Aspac1/Aspac1.home.html), as well as an internal contaminant database, using X!Tandem Cyclone software (X!Tandem, Jouy-en-Josas, France). The protein annotation was completed manually by BlastP using the NCBI nr database (https://blast.ncbi.nlm.nih.gov) and the CAZy families were annotated using the CAZy database.

### Saccharification assays with secretomes

Wheat straw, Miscanthus and poplar were obtained from IFP Energies Nouvelles (Rueil-Malmaison, France). Biomass were pre-treated by steam explosion under acidic conditions, washed in hot water to remove free products, and dried at 55 °C. After 1 week at room temperature, they were shredded and sieved to a 0.8-mm maximal size.

The reference K975 cocktail, supplied by IFP Energies Nouvelles, was obtained by growing *T. reesei* strain CL847 in the presence of lactose, and has a specific β-glucosidase activity of 0.8 IU/mg. It was complemented by SP188, a commercial cocktail of β-*Aspergillus niger* glucosidases (Novozyme, Bagsværd, Denmark).

Saccharification tests were performed in 96-well plates using 5% (w/v) biomass in 50 mM sodium acetate buffer (pH 4.8) containing 0.1 g L^−1^ chloramphenicol, with a K975 enzyme loading of 5 mg g^−1^ of dry matter (DM) and a total β-glucosidase activity of 80 UI/g DM. 10 µL of 1:10 diluted secretomes were added, replaced by 10 µL of buffer for the reference conditions. Each microplate contained seven replicates of each condition, as well as control conditions with biomass alone and enzymes alone. The sealed plates were incubated at 45 °C under rotary agitation at 850 rpm for 24–96 h. At each sampling time, plates were centrifuged and the supernatant was filtered and stored at − 20 °C. Glucose concentration was measured using the Glucose GOD-PAP reagent (Biolabo, Maizy, France) using a standard glucose range and yields were calculated taking into account the initial amount of cellulosic glucose.

Between 7 and 30 independent reactions were performed for each secretome. To combine the results of these assays, the saccharification yields obtained in the presence of secretomes were translated into a percentage of improvement compared to the internal reference of each assay. For each condition, a Student *t*-test was performed to determine if the mean of all the replicates was statistically different from the mean of the reference values, using the *p* value as a criterion.

### Bioinformatic analysis of the AA16 family

208 sequences containing a X273 module were chosen from genomes in the NCBI or the JGI Mycocosm. To avoid interference from the presence or the absence of additional residues or domains, the signal peptides and C-terminal extensions were removed prior to the alignment. The resulting amino acid sequences corresponding to the catalytic domain were aligned using MUSCLE [52], operating with default parameters. A distance matrix was derived from the alignment using Blosum62 substitution parameters [53] and subsequently used to build a phylogenetic tree using an improved version of the neighbor-joining method [54]. The tree was displayed using Dendroscope [55], and the visualization of consensus amino acid sequence of the catalytic module was made using WebLogo [56].

### Cloning and production of enzymes

The nucleotide sequence coding for the AA16 of *A. aculeatus* (Genebank ID XP_020060743.1) was codon optimized for *Pichia pastoris*. The region corresponding to the native signal sequence was kept, and the C-terminal extension region was removed. Total synthesis of the genes was performed by Genewiz (South Plainfield, New-Jersey, USA) and the genes were inserted in the expression vector pPICZαA (Invitrogen, Carlsbad, California, USA) in frame with the C-terminal poly-histidine tag. Transformation of competent *P. pastoris* SuperMan_5_ cells (BioGrammatics, Carlsbad, California, USA) was performed by electroporation with PmeI-linearized pPICZαA recombinant plasmids as described in [57]. Zeocin-resistant transformants were then screened for protein production.

The best-producing transformants were grown in 2 L of BMGY media containing 1 mL L^−1^ of Pichia trace minerals 4 (PTM4) salts (2 g L^−1^ CuSO_4_·5H_2_O, 3 g L^−1^ MnSO_4_·H_2_O, 0.2 g L^−1^ Na_2_MoO_4_·2H_2_O, 0.02 g L^−1^ H_3_BO_3_, 0.5 g L^−1^ CaSO_4_·2H_2_O, 0.5 g L^−1^ CoCl_2_, 12.5 g L^−1^ ZnSO_4_·7H_2_O, 22 g L^−1^ FeSO_4_·7H_2_O, NaI 0.08 g L^−1^, H_2_SO_4_ 1 mL L^−1^) and 0.2 g L^−1^ of biotin in flasks at 30 °C in an orbital shaker (200 rpm) for 16 h, to an OD_600_ of 2 to 6. Expression was induced by transferring cells into 400 mL of BMMY media containing 1 mL L^−1^ of PTM4 salts at 20 °C in an orbital shaker (200 rpm) for another 3 days. Each day, the medium was supplemented with 3% (v/v) methanol. The cells were harvested by centrifugation, and just before purification, the supernatant had its pH adjusted to 7.8 and was filtrated on 0.45-µm membrane (Millipore, Burlington, Massachusetts, USA).

Bioreactor production was carried out in a 1.3-L New Brunswick BioFlo 115 fermentor (Eppendorf, Hamburg, Germany) following the *P. pastoris* fermentation process guidelines (Invitrogen), replacing the glycerol fed-batch phase by a 4-h sorbitol and methanol transition phase.

### Purification by affinity chromatography

Filtered culture supernatant was loaded onto a 5-mL HisTrap HP column (GE Healthcare, Bus, France) equilibrated with buffer A (Tris–HCl 50 mM pH 7.8, NaCl 150 mM, imidazole 10 mM) that was connected to an Äkta purifier 100 (GE Healthcare). (His)_6_-tagged recombinant proteins were eluted with buffer B (Tris–HCl 50 mM pH 7.8, NaCl 150 mM, imidazole 500 mM). Fractions containing recombinant enzymes were pooled, concentrated, and dialyzed against sodium acetate buffer 50 mM, pH 5.2. A fraction of eluate was loaded onto 10% Tris–glycine precast SDS-PAGE gels (BioRad) that were stained with Coomassie Blue. The protein concentrations were determined by absorption at 280 nm using a Nanodrop ND-2000 spectrophotometer (Thermo Fisher Scientific) and theoretical molecular weights and molar extinction coefficient.

### Purification by ion exchange chromatography

Filtered culture supernatant was loaded onto a 20-mL HiPrep DEAE FF 16/10 column (GE Healthcare), equilibrated with Tris–HCl 50 mM pH 7.8. Proteins were eluted using a linear gradient of 1 M NaCl (0 to 500 mM in 180 mL). Fractions containing recombinant proteins were pooled and concentrated. A fraction of eluate was loaded onto 10% Tris–glycine precast SDS-PAGE gels (BioRad) that were stained with Coomassie Blue. The protein concentrations were determined by absorption at 280 nm using a Nanodrop ND-2000 spectrophotometer (Thermo Fisher Scientific) and theoretical molecular weights and molar extinction coefficient.

### N-terminal amino acid sequence determination

The N-terminal amino acid sequences of purified AaAA16 were determined according to the Edman degradation. Samples were electroblotted onto a polyvinylidene difluoride membrane (iBlot, Thermo Fisher Scientific). Analyses were carried out on a Procise Sequencing System (Thermo Fisher Scientific).

### ICP-MS analysis

The ICP-MS analysis was performed as described in [18]. The samples were mineralized, then diluted in ultrapure water, and analyzed by an ICAP Q apparatus (Thermo Electron, Les Ullis, France). The copper concentration was determined using Plasmalab (Thermo Electron) software, at m/z = 63.

### Amplex Red assay

A fluorimetric assay based on Amplex Red and horseradish peroxidase was used as described previously [44]. 96 black well plates (Greiner Bio One, Kremsmünster, Austria) containing 50 mM citrate–phosphate buffer (pH 6), 50 µM Amplex Red (Sigma-Aldrich), 7.1 U mL^−1^ horseradish peroxidase, 10 µM LPMOs and 50 µM reducing agent (l-cysteine or ascorbate) in a total volume of 100 µL were incubated at 30 °C for 30 min. Fluorescence was followed at a rate of one point per minute, at an excitation wavelength of 560 nm and an emission wavelength of 595 nm, using a Tecan Infinite M200 (Tecan, Männedorf, Switzerland) plate reader.

### Substrates cleavage assays

Cleavage assays were performed in a 300-µL volume of water containing 0.1% (m/v) of solid substrates (PASC, Avicel, barley β-1,3/1,4-glucan, konjac glucomannan) or 1 mM of soluble substrates (cellohexaose, xylohexaose, xyloglucan oligosaccharides of known structure XXXG, XXLG, and XLLG according to the nomenclature of Fry et al. [58]). Avicel was purchased from Sigma-Aldrich, PASC was prepared from Avicel according to the method described by Wood et al. [59], and the other products were purchased from Megazyme (Bray, Ireland).

Reaction media contained AaAA16 (4.4–20 µM) and 1 mM l-cysteine. *Podospora anserina* cellobiose dehydrogenase *Pa*CDHB [11] (2 nM) was also used as an enzymatic electron donor. Samples were incubated for 24 h in a thermomixer (Eppendorf, Montesson, France) at 45 °C and 850 rpm. The soluble products were separated from the insoluble fraction reaction by centrifuging the samples at 15,000*g* for 10 min.

### Synergy assays

Synergy assays between AaAA16 and *T. reesei* CBHI were performed in a total volume of 800 µL containing 0.1% (m/v) PASC or NFC in 50 mM pH 5.2 acetate buffer with 8 µg AaAA16 and 1 mM l-cysteine. The samples were incubated in triplicates in a thermomixer (Eppendorf) at 45 °C and 850 rpm, for 24 h. The samples were then boiled for at least 10 min and centrifuged at 15,000*g* for 10 min. The supernatant was removed, and the remaining insoluble fraction of the substrate was washed in buffer twice. Hydrolysis by *T. reesei* CBHI (0.8 µg) was performed in 800 µL of 50 mM pH 5.2 acetate buffer for 2 h at 45 °C and 850 rpm. The reaction was stopped as described above, and the soluble and insoluble fractions were separated.

### Analysis of oxidized and non-oxidized oligosaccharides

Mono- and oligosaccharides in the soluble fractions resulting from substrate cleavage and synergy assays were detected by high-performance anion-exchange chromatography (HPAEC) coupled with pulsed amperometric detection (PAD) (Dionex, Thermo Fisher Scientific), according to the method described by Westereng et al. [60] using non-oxidized cello-oligosaccharides (DP2–DP6) as standards (Megazyme). Briefly, the eluents were 0.1 M NaOH (eluent A) and 1 M NaOAc in 0.1 M NaOH (eluent B). Elution was performed at a constant flow rate of 0.25 mL/min at 30 °C, using a linear gradient of 0–10% eluent B over 10 min, 10–30% eluent B over 25 min, and an exponential gradient of 30–100% eluent B in 5 min. The initial condition (100% eluent A) was then restored in 1 min and maintained for 9 min to recondition the column.

Mass spectrometry experiment (MS/MS) were performed on a Synapt G2Si high-definition mass spectrometer (Waters Corp., Manchester, UK) equipped with an Electrospray ion (ESI) source. After the ion precursor selection and prior the fragmentation step, ion mobility (IM) was activated to reduce interference from sample impurities. IM was performed in a traveling-wave ion mobility (TWIM) cell. Helium flows was held at 180 mL min^−1^ in the helium cell and nitrogen flow was adjusted at 90 mL min^−1^ in the mobility cell. The IM traveling wave height was set to 40 V, and its wave velocity was set to 300 m s^−1^. After passing through the mobility cell, oxidized species were fragmented by collision-induced dissociation in the transfer cell of the instrument (MS/MS). In these experiments, samples were diluted tenfold in MeOH/H_2_O (1:1, v/v) and infused at a flow rate of 5 μL min^−1^. Acquisitions were conducted in negative polarity, as well as in ‘sensitivity’ mode.

## Additional file

**Additional file 1:** **Table S1.** Protein concentration (determined by BCA assay) of the secretomes of five *Aspergillus* spp. strains grown on three inducers, after seven days of culture. **Figure S1.** Hydrolysis of three pretreated lignocellulosic substrates by a cellulolytic cocktail of *Trichoderma reesei* strain CL847 produced on lactose. Error bars represent standard deviations calculated on 3 replicates. **Figure S2.** Graphical representation of AA16 module consensus amino acids, based on the alignment of 213 sequences, generated using the WebLogo application [1]. The strictly conserved histidine residues are shown in positions 1 and 107. **Figure S3.** Time profiles of dissolved oxygen (DO), temperature and optical density (OD) at 600 nm during the production of AaAA16 by *Pichia pastoris* in a 1.3-L bioreactor. 1: Glycerol batch phase; 2: Sorbitol and methanol transition phase; 3: Methanol fed-batch phase. **Figure S4.** HPAEC–PAD chromatograms of 0.1% PASC soluble degradation products, after 70 h incubation with *P. pastoris* bioreactor supernatant (SN, blue chromatogram) or with AaAA16 protein purified by affinity chromatography using a nickel HisTrap column (black chromatogram). **Figure S5.** SDS-PAGE analysis of AaAA16 before and after purification. Lane A, *Pichia pastoris* bioreactor supernatant; lane B, AaAA16 purified by ion exchange chromatography; lane M, Molecular weight protein ladder. **Figure S6.** Production of H_2_O_2_ by AaAA16 in the presence of ascorbate or l-cysteine, visualized using the Amplex red coupled assay. The produced H_2_O_2_ is used by horse radish peroxidase to transform Amplex Red into fluorescent resorufin, which is measured by fluorimetric counts at 560 nm. **Figure S7.** HPAEC–PAD chromatograms showing soluble products generated from 0.5 mM cellohexaose (G6) using 4.4 µM AaAA16 and 1 mM l-cysteine. The LPMO9H from *Podospora anserina* (PaAA9H) [2] was used as a reference of LPMO acting on cello-oligosaccharides (producing native sugars and C4-oxidized products, visible at 26 and 40 min in the box at the top right). **Figure S8.** HPAEC–PAD chromatograms of PASC soluble degradation products, after incubation with AaAA16 alone or using *Podospora anserina* cellobiodehydrogenase (PaCDHB) [2] or l-cysteine as electron donors. The oxidized products visible in the CDH condition (green chromatogram) are the result of the action of CDH on cellodextrins released from PASC by AaAA16 (blue chromatogram). The proper LPMO activity is only visible after activation by l-cysteine (red chromatogram).

## Abbreviations

AA: auxiliary activity enzyme
Avi: Avicel
BCA: bicinchoninic acid
CAZyme: carbohydrate-active enzyme
CBH: cellobiohydrolase
CBM: carbohydrate binding module
CDH: cellobiose dehydrogenase
CE: carbohydrate esterase
DM: dry matter
DP: degree of polymerization
ESI-MS: electrospray ionisation mass spectrometry
GH: glycoside hydrolase
GPI: glycosylphosphatidylinositol
HMM: Hidden Markov model
HPAEC–PAD: high-performance anion-exchange chromatography coupled with amperometric detection
ICP-MS: inductively coupled plasma mass spectrometry
IMAC: immobilized metal ion affinity chromatography
LC–MS/MS: liquid chromatography coupled to tandem mass spectrometry
LPMO: lytic polysaccharide monooxygenase
MB: maize bran
NFC: nanofibrillated cellulose
OD_600_: absorbance at 600 nm
PASC: phosphoric acid swollen cellulose
PL: polysaccharide lyase
SBP: sugarbeet pulp
SDS-PAGE: sodium dodecyl sulfate-polyacrylamide gel electrophoresis
SP: signal peptide

## References

[1] de Bhowmick G, Sarmah AK, Sen R (2018). Lignocellulosic biorefinery as a model for sustainable development of biofuels and value added products. Biores Technol.

[2] Cherubini F (2010). The biorefinery concept: using biomass instead of oil for producing energy and chemicals. Energy Convers Manage.

[3] Lombard V, Golaconda Ramulu H, Drula E, Coutinho PM, Henrissat B (2014). The carbohydrate-active enzymes database (CAZy) in 2013. Nucleic Acids Res.

[4] Vaaje-Kolstad G, Westereng B, Horn SJ, Liu Z, Zhai H, Sørlie M, Eijsink VGH (2010). An oxidative enzyme boosting the enzymatic conversion of recalcitrant polysaccharides. Science.

[5] Harris PV, Welner D, McFarland KC, Re E, Navarro Poulsen J-C, Brown K (2010). Stimulation of lignocellulosic biomass hydrolysis by proteins of glycoside hydrolase family 61: structure and function of a large, enigmatic family. Biochemistry.

[6] Bissaro B, Várnai A, Røhr ÅK, Eijsink VGH (2018). oxidoreductases and reactive oxygen species in conversion of lignocellulosic biomass. Microbiol Mol Biol Rev.

[7] Bissaro B, Rohr AK, Muller G, Chylenski P, Skaugen M, Forsberg Z (2017). Oxidative cleavage of polysaccharides by monocopper enzymes depends on H2O2. Nat Chem Biol.

[8] Cannella D, Möllers KB, Frigaard N-U, Jensen PE, Bjerrum MJ, Johansen KS, Felby C (2016). Light-driven oxidation of polysaccharides by photosynthetic pigments and a metalloenzyme. Nat Commun..

[9] Quinlan RJ, Sweeney MD, Lo Leggio L, Otten H, Poulsen JCN, Johansen KS (2011). Insights into the oxidative degradation of cellulose by a copper metalloenzyme that exploits biomass components. Proc Natl Acad Sci USA.

[10] Ciano L, Davies GJ, Tolman WB, Walton PH (2018). Bracing copper for the catalytic oxidation of C-H bonds. Nature Catalysis..

[11] Bennati-Granier C, Garajova S, Champion C, Grisel S, Haon M, Zhou S (2015). Substrate specificity and regioselectivity of fungal AA9 lytic polysaccharide monooxygenases secreted by Podospora anserina. Biotechnol Biofuels.

[12] Isaksen T, Westereng B, Aachmann FL, Agger JW, Kracher D, Kittl R (2014). A C4-oxidizing lytic polysaccharide monooxygenase cleaving both cellulose and cello-oligosaccharides. J Biol Chem.

[13] Fanuel M, Garajova S, Ropartz D, McGregor N, Brumer H, Rogniaux H, Berrin J-G (2017). The Podospora anserina lytic polysaccharide monooxygenase PaLPMO9H catalyzes oxidative cleavage of diverse plant cell wall matrix glycans. Biotechnol Biofuels.

[14] Vaaje-Kolstad G, Bøhle LA, Gåseidnes S, Dalhus B, Bjørås M, Mathiesen G, Eijsink VGH (2012). Characterization of the chitinolytic machinery of *Enterococcus faecalis* V583 and high-resolution structure of its oxidative CBM33 enzyme. J Mol Biol.

[15] Hemsworth GR, Henrissat B, Davies GJ, Walton PH (2014). Discovery and characterization of a new family of lytic polysaccharide monooxygenases. Nat Chem Biol.

[16] Lo Leggio L, Simmons TJ, Poulsen JCN, Frandsen KEH, Hemsworth GR, Stringer MA (2015). Structure and boosting activity of a starch-degrading lytic polysaccharide monooxygenase. Nat Commun..

[17] van Vu V, Marletta MA (2016). Starch-degrading polysaccharide monooxygenases. Cell Mol Life Sci.

[18] Couturier M, Ladevèze S, Sulzenbacher G, Ciano L, Fanuel M, Moreau C (2018). Lytic xylan oxidases from wood-decay fungi unlock biomass degradation. Nature Chemical Biology.

[19] Sabbadin F, Hemsworth GR, Ciano L, Henrissat B, Dupree P, Tryfona T (2018). An ancient family of lytic polysaccharide monooxygenases with roles in arthropod development and biomass digestion. Nat Commun..

[20] Johansen KS (2016). Discovery and industrial applications of lytic polysaccharide mono-oxygenases. Biochem Soc Trans.

[21] Peterson R, Nevalainen H (2012). *Trichoderma reesei* RUT-C30—thirty years of strain improvement. Microbiology.

[22] Seidl V, Seiboth B (2010). *Trichoderma reesei*: genetic approaches to improving strain efficiency. Biofuels..

[23] Häkkinen M, Arvas M, Oja M, Aro N, Penttilä M, Saloheimo M, Pakula TM (2012). Re-annotation of the CAZy genes of *Trichoderma reesei* and transcription in the presence of lignocellulosic substrates. Microb Cell Fact.

[24] Martinez D, Berka RM, Henrissat B, Saloheimo M, Arvas M, Baker SE (2008). Genome sequencing and analysis of the biomass-degrading fungus *Trichoderma reesei* (syn. Hypocrea jecorina). Nat Biotechnol.

[25] Bischof RH, Ramoni J, Seiboth B (2016). Cellulases and beyond: the first 70 years of the enzyme producer *Trichoderma reesei*. Microb Cell Fact.

[26] Lenfant N, Hainaut M, Terrapon N, Drula E, Lombard V, Henrissat B (2017). A bioinformatics analysis of 3400 lytic polysaccharide oxidases from family AA9. Carbohydr Res.

[27] Bouws H, Wattenberg A, Zorn H (2008). Fungal secretomes—nature’s toolbox for white biotechnology. Appl Microbiol Biotechnol.

[28] Di Cologna NDM, Gómez-Mendoza DP, Zanoelo FF, Giannesi GC, Guimarães NCDA, Moreira LRDS (2017). Exploring Trichoderma and Aspergillus secretomes: proteomics approaches for the identification of enzymes of biotechnological interest. Enzyme Microbial Technol.

[29] Berrin J-G, Rosso M-N, Abou Hachem M (2017). Fungal secretomics to probe the biological functions of lytic polysaccharide monooxygenases. Carbohydr Res.

[30] Nekiunaite L, Arntzen MØ, Svensson B, Vaaje-Kolstad G, Abou Hachem M (2016). Lytic polysaccharide monooxygenases and other oxidative enzymes are abundantly secreted by *Aspergillus nidulans* grown on different starches. Biotechnol Biofuels.

[31] Berrin J-G, Navarro D, Lopes-Ferreira N, Margeot A, Coutinho P, Henrissat B. Multi-enzymatic preparation containing the secretome of an Aspergillus japonicus strain (WO2014037925A1); 2015.

[32] Herpoël-Gimbert I, Margeot A, Dolla A, Jan G, Mollé D, Lignon S (2008). Comparative secretome analyses of two *Trichoderma reesei* RUT-C30 and CL847 hypersecretory strains. Biotechnol Biofuels.

[33] Durand H, Clanet M, Tiraby G (1988). Genetic improvement of *Trichoderma reesei* for large scale cellulase production. Enzyme Microb Technol.

[34] Abarca ML, Accensi F, Cano J, Cabañes FJ (2004). Taxonomy and significance of black aspergilli. Antonie Van Leeuwenhoek.

[35] Voshol GP, Vijgenboom E, Punt PJ (2017). The discovery of novel LPMO families with a new Hidden Markov model. BMC Res Notes..

[36] Butler MI, Gray J, Goodwin TJD, Poulter RTM (2006). The distribution and evolutionary history of the PRP8 intein. BMC Evol Biol.

[37] van Vu V, Beeson WT, Span EA, Farquhar ER, Marletta MA (2014). A family of starch-active polysaccharide monooxygenases. Proc Natl Acad Sci U S A..

[38] Kim K, Rhee SG, Stadtman ER (1985). Nonenzymatic cleavage of proteins by reactive oxygen species generated by dithiothreitol and iron. J Biol Chem.

[39] McMahon HE, Mangé A, Nishida N, Créminon C, Casanova D, Lehmann S (2001). Cleavage of the amino terminus of the prion protein by reactive oxygen species. J Biol Chem.

[40] Loose JSM, Arntzen MØ, Bissaro B, Ludwig R, Eijsink VGH, Vaaje-Kolstad G (2018). Multipoint precision binding of substrate protects lytic polysaccharide monooxygenases from self-destructive off-pathway processes. Biochemistry.

[41] Uchida K, Kawakishi S (1989). Ascorbate-mediated specific oxidation of the imidazole ring in a histidine derivative. Bioorg Chem.

[42] Stadtman ER (1993). Oxidation of free amino acids and amino acid residues in proteins by radiolysis and by metal-catalyzed reactions. Annu Rev Biochem.

[43] Urresti S, Cartmell A, Liu F, Walton PH, Davies GJ (2018). Structural studies of the unusual metal-ion site of the GH124 endoglucanase from Ruminiclostridium thermocellum. Acta Crystallogr F Struct Biol Commun..

[44] Kittl R, Kracher D, Burgstaller D, Haltrich D, Ludwig R (2012). Production of four Neurospora crassa lytic polysaccharide monooxygenases in Pichia pastoris monitored by a fluorimetric assay. Biotechnol Biofuels.

[45] Selig MJ, Vuong TV, Gudmundsson M, Forsberg Z, Westereng B, Felby C, Master ER (2015). Modified cellobiohydrolase–cellulose interactions following treatment with lytic polysaccharide monooxygenase CelS2 (ScLPMO10C) observed by QCM-D. Cellulose.

[46] Song B, Li B, Wang X, Shen W, Park S, Collings C (2018). Real-time imaging reveals that lytic polysaccharide monooxygenase promotes cellulase activity by increasing cellulose accessibility. Biotechnol Biofuels.

[47] Langston JA, Shaghasi T, Abbate E, Xu F, Vlasenko E, Sweeney MD (2011). Oxidoreductive cellulose depolymerization by the enzymes cellobiose dehydrogenase and glycoside hydrolase 61. Appl Environ Microbiol.

[48] Eibinger M, Ganner T, Bubner P, Rošker S, Kracher D, Haltrich D (2014). Cellulose surface degradation by a lytic polysaccharide monooxygenase and its effect on cellulase hydrolytic efficiency. J Biol Chem.

[49] Payne CM, Knott BC, Mayes HB, Hansson H, Himmel ME, Sandgren M (2015). Fungal cellulases. Chem Rev.

[50] Petrovi DM, Bissaro B, Chylenski P, Skaugen M, Sørlie M, Jensen MS (2018). Methylation of the N-terminal histidine protects a lytic polysaccharide monooxygenase from auto-oxidative inactivation. Protein Sci.

[51] Navarro D, Couturier M, Damasceno da Silva GG, Berrin J-G, Rouau X, Asther M, Bignon C (2010). Automated assay for screening the enzymatic release of reducing sugars from micronized biomass. Microbial Cell Fact.

[52] Edgar RC (2004). MUSCLE: multiple sequence alignment with high accuracy and high throughput. Nucleic Acids Res.

[53] Henikoff S, Henikoff JG (1992). Amino acid substitution matrices from protein blocks. Proc Natl Acad Sci USA.

[54] Gascuel O (1997). BIONJ: an improved version of the NJ algorithm based on a simple model of sequence data. Mol Biol Evol.

[55] Huson DH, Scornavacca C (2012). Dendroscope 3: an interactive tool for rooted phylogenetic trees and networks. Syst Biol.

[56] Crooks GE, Hon G, Chandonia J-M, Brenner SE (2004). WebLogo: a sequence logo generator. Genome Res.

[57] Couturier M, Haon M, Coutinho PM, Henrissat B, Lesage-Meessen L, Berrin J-G (2011). Podospora anserina hemicellulases potentiate the *Trichoderma reesei* secretome for saccharification of lignocellulosic biomass. Appl Environ Microbiol.

[58] Fry SC, York WS, Albersheim P, Darvill A, Hayashi T, Joseleau J-P (1993). An unambiguous nomenclature for xyloglucan-derived oligosaccharides. Physiol Plant.

[59] Wood TM. Preparation of crystalline, amorphous, and dyed cellulase substrates. In: Methods in enzymology. Academic Press; 1988. p. 19–25. 10.1016/0076-6879(88)60103-0.

[60] Westereng B, Agger JW, Horn SJ, Vaaje-Kolstad G, Aachmann FL, Stenstrøm YH, Eijsink VGH (2013). Efficient separation of oxidized cello-oligosaccharides generated by cellulose degrading lytic polysaccharide monooxygenases. J Chromatogr A.

