# Supplementary material for: AA16, a new lytic polysaccharide monooxygenase family identified in fungal secretomes
Source: Biotechnol Biofuels. 2019 Mar 16;12:55. doi: 10.1186/s13068-019-1394-y (PMC6420742; doi:10.1186/s13068-019-1394-y)
Supplement: Supplementary file 1 — Additional file 1: Table S1. Protein concentration (determined by BCA assay) of the secretomes of five Aspergillus spp. strains grown on three inducers, after seven days of culture. Figure S1. Hydrolysis of three pretreated lignocellulosic substrates by a cellulolytic cocktail of Trichoderma reesei strain CL847 produced on lactose. Error bars represent standard deviations calculated on 3 replicates. Figure S2. Graphical representation of AA16 module consensus amino acids, based on the alignment of 213 sequences, generated using the WebLogo application [1]. The strictly conserved histidine residues are shown in positions 1 and 107. Figure S3. Time profiles of dissolved oxygen (DO), temperature and optical density (OD) at 600 nm during the production of AaAA16 by Pichia pastoris in a 1.3-L bioreactor. 1: Glycerol batch phase; 2: Sorbitol and methanol transition phase; 3: Methanol fed-batch phase. Figure S4. HPAEC–PAD chromatograms of 0.1% PASC soluble degradation products, after 70 h incubation with P. pastoris bioreactor supernatant (SN, blue chromatogram) or with AaAA16 protein purified by affinity chromatography using a nickel HisTrap column (black chromatogram). Figure S5. SDS-PAGE analysis of AaAA16 before and after purification. Lane A, Pichia pastoris bioreactor supernatant; lane B, AaAA16 purified by ion exchange chromatography; lane M, Molecular weight protein ladder. Figure S6. Production of H2O2 by AaAA16 in the presence of ascorbate or l-cysteine, visualized using the Amplex red coupled assay. The produced H2O2 is used by horse radish peroxidase to transform Amplex Red into fluorescent resorufin, which is measured by fluorimetric counts at 560 nm. Figure S7. HPAEC–PAD chromatograms showing soluble products generated from 0.5 mM cellohexaose (G6) using 4.4 µM AaAA16 and 1 mM l-cysteine. The LPMO9H from Podospora anserina (PaAA9H) [2] was used as a reference of LPMO acting on cello-oligosaccharides (producing native sugars and C4-oxidized products, visible a [file 13068_2019_1394_MOESM1_ESM.docx]

**AA16, a new Lytic Polysaccharide Monooxygenase family identified in fungal secretomes**

Camille Filiatrault Chastel, David Navarro_,_  Mireille Haon, Sacha Grisel, Isabelle Herpoël-Gimbert, Didier Chevret, Mathieu Fanuel, Bernard Henrissat, Senta Heiss-Blanquet, Antoine Margeot and Jean-Guy Berrin.

**Additional data**

**Table S1:** Protein concentration (determined by BCA assay) of the secretomes of five *Aspergillus* spp. strains grown on three inducers, after seven days of culture.

| Code | Species | Strain number | Inducer | Concentration (mg/mL) |
| --- | --- | --- | --- | --- |
| 405_Avi | *Aspergillus japonicus* | CIRM-BRFM 405 | Avicel | 0.55 |
| 405_MB | *Aspergillus japonicus* | CIRM-BRFM 405 | Maize bran | 12.56 |
| 405_SBP | *Aspergillus japonicus* | CIRM-BRFM 405 | Sugar beet pulp | 4.96 |
| 430_Avi | *Aspergillus niger* | CIRM-BRFM 430 | Avicel | 0.27 |
| 430_MB | *Aspergillus niger* | CIRM-BRFM 430 | Maize bran | 17.63 |
| 430_SBP | *Aspergillus niger* | CIRM-BRFM 430 | Sugar beet pulp | 7.98 |
| 1487_Avi | *Aspergillus japonicus* | CIRM-BRFM 1487 | Avicel | 0.25 |
| 1487_MB | *Aspergillus japonicus* | CIRM-BRFM 1487 | Maize bran | 17.24 |
| 1487_SBP | *Aspergillus japonicus* | CIRM-BRFM 1487 | Sugar beet pulp | 8.43 |
| 1489_Avi | *Aspergillus japonicus* | CIRM-BRFM 1489 | Avicel | 0.29 |
| 1489_MB | *Aspergillus japonicus* | CIRM-BRFM 1489 | Maize bran | 13.47 |
| 1489_SBP | *Aspergillus japonicus* | CIRM-BRFM 1489 | Sugar beet pulp | 12.37 |
| 1490_Avi | *Aspergillus japonicus* | CIRM-BRFM 1490 | Avicel | 0.34 |
| 1490_MB | *Aspergillus japonicus* | CIRM-BRFM 1490 | Maize bran | 12.31 |
| 1490_SBP | *Aspergillus japonicus* | CIRM-BRFM 1490 | Sugar beet pulp | 8.50 |

**Figure S1:** Hydrolysis of three pretreated lignocellulosic substrates by a cellulolytic cocktail of *Trichoderma reesei* strain CL847 produced on lactose. Error bars represent standard deviations calculated on 3 replicates.

**Figure S2:** Graphical representation of AA16 module consensus amino acids, based on the alignment of 213 sequences, generated using the WebLogo application [[1]](#_CTVL0014a860a27b8654ce19bbf3bf9d420398f). The strictly conserved histidine residues are shown in positions 1 and 107.


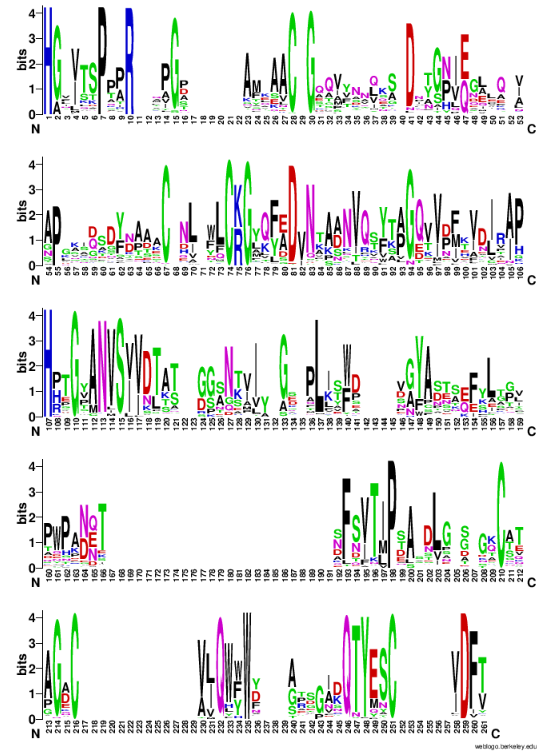


**Figure S3:** Time profiles of dissolved oxygen (DO), temperature and optical density (OD) at 600 nm during the production of AaAA16 by *Pichia pastoris* in a 1.3-L bioreactor. 1: Glycerol batch phase; 2 : Sorbitol and methanol transition phase; 3 : Methanol fed-batch phase.

_① ② ③_

**Figure S4:** HPAEC-PAD chromatograms of 0.1% PASC soluble degradation products, after 70h incubation with *P. pastoris* bioreactor supernatant (SN, blue chromatogram) or with AaAA16 protein purified by affinity chromatography using a nickel HisTrap column (black chromatogram).


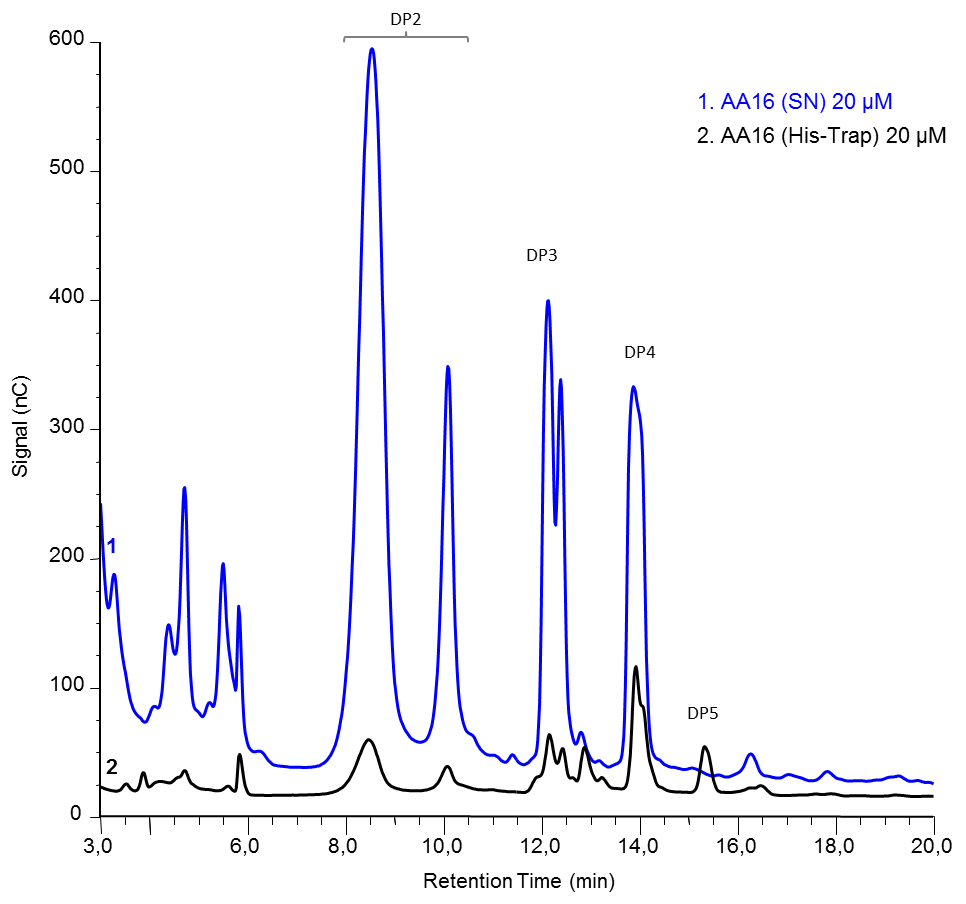


**Figure S5:** SDS-PAGE analysis of AaAA16 before and after purification. Lane A, *Pichia pastoris* bioreactor supernatant; lane B, AaAA16 purified by ion exchange chromatography; lane M, Molecular weight protein ladder.


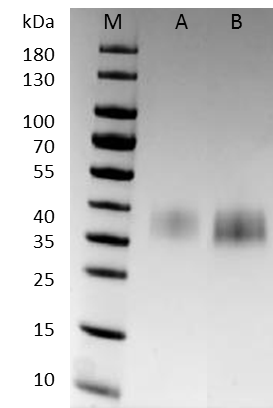


**Figure S6:** Production of H_2_O_2_ by AaAA16 in the presence of ascorbate or L-cysteine, visualized using the Amplex red coupled assay. The produced H_2_O_2_ is used by horse radish peroxidase to transform Amplex Red into fluorescent resorufin, which is measured by fluorimetric counts at 560 nm.


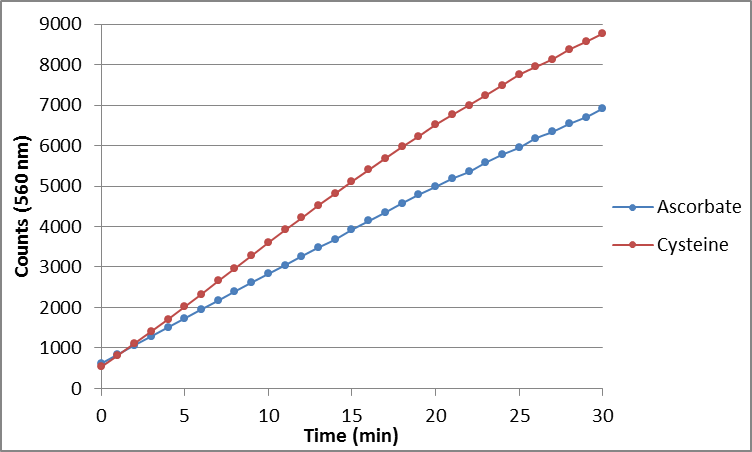


**Figure S7:** HPAEC-PAD chromatograms showing soluble products generated from 0.5 mM cellohexaose (G6) using 4.4 µM AaAA16 and 1 mM L-cysteine. The LPMO9H from *Podospora anserina* (PaAA9H) [[2]](#_CTVL001cbe12a7f57744f25abb2c7a14896e88b) was used as a reference of LPMO acting on cello-oligosaccharides (producing native sugars and C4-oxidized products, visible at 26 and 40 min in the box at the top right).

**
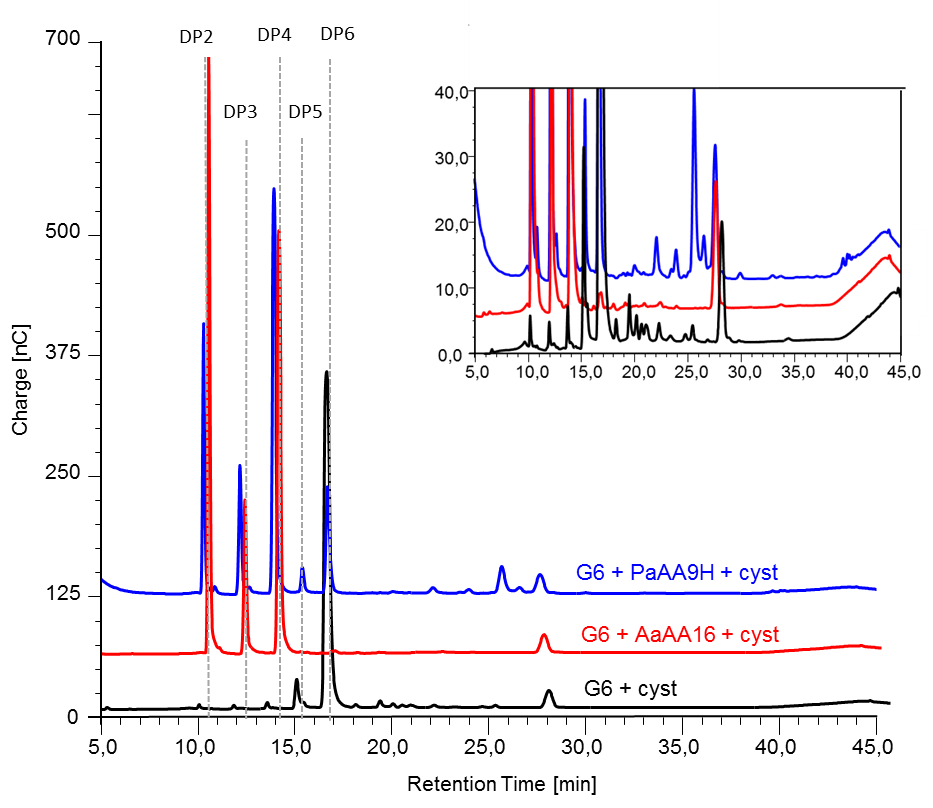
**

**Figure S8:** HPAEC-PAD chromatograms of PASC soluble degradation products, after incubation with AaAA16 alone or using *Podospora anserina* cellobiodehydrogenase (PaCDHB) [[2]](#_CTVL001cbe12a7f57744f25abb2c7a14896e88b) or L-cysteine as electron donors. The oxidized products visible in the CDH condition (green chromatogram) are the result of the action of CDH on cellodextrins released from PASC by AaAA16 (blue chromatogram). The proper LPMO activity is only visible after activation by L-cysteine (red chromatogram).


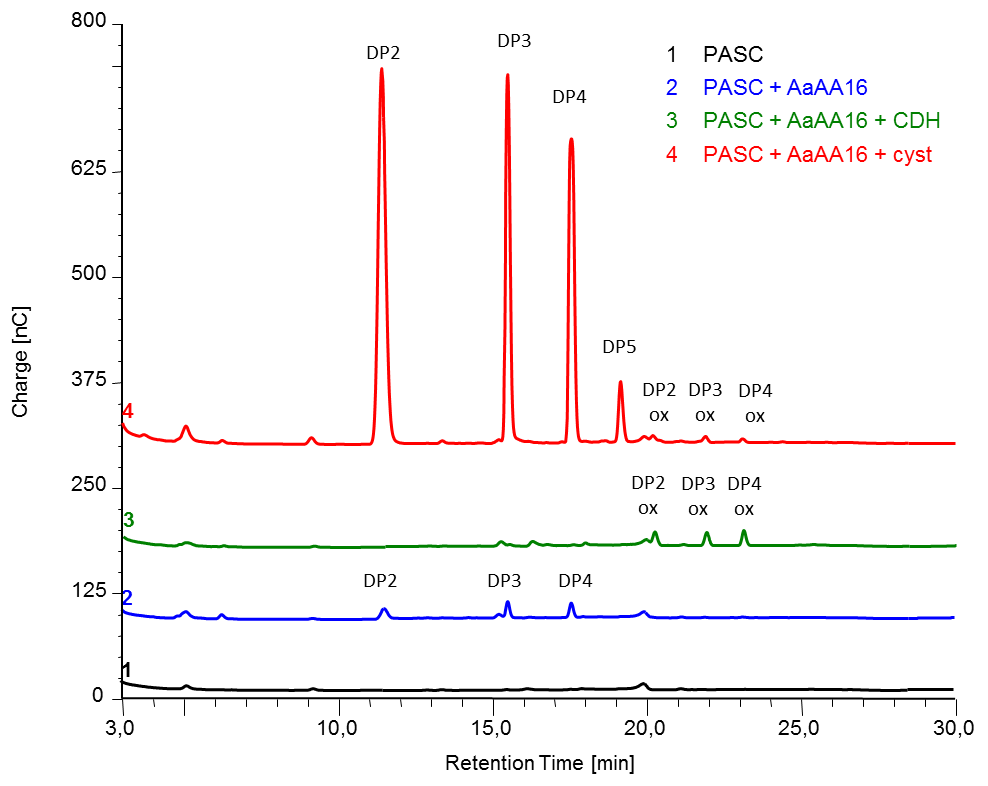


References

1. Crooks GE, Hon G, Chandonia J-M, Brenner SE. WebLogo: A Sequence Logo Generator. Genome Res. 2004;14:1188–90.

2. Bennati-Granier C, Garajova S, Champion C, Grisel S, Haon M, Zhou S, et al. Substrate specificity and regioselectivity of fungal AA9 lytic polysaccharide monooxygenases secreted by Podospora anserina. Biotechnol Biofuels. 2015;8:90.
